# Clinical Anatomy and Medical Malpractice—A Narrative Review with Methodological Implications

**DOI:** 10.3390/healthcare10101915

**Published:** 2022-09-30

**Authors:** Andrea Porzionato, Veronica Macchi, Carla Stecco, Rafael Boscolo-Berto, Marios Loukas, Ronald Shane Tubbs, Raffaele De Caro

**Affiliations:** 1 Section of Anatomy, Department of Neuroscience, University of Padova, Via Gabelli, 65, 35127 Padova, Italy; 2 Department of Anatomical Sciences, True Blue Campus, St. George’s University, St. George 1473, Grenada; 3 Department of Neurosurgery, Tulane University, New Orleans, LA 70112, USA

**Keywords:** medical malpractice, anatomical variations, iatrogenic lesions, post-mortem imaging, clinical anatomy, forensic clinical anatomy, medical responsibility, medical liability, individual anatomy

## Abstract

Anatomical issues are intrinsically included in medico-legal methodology, however, higher awareness would be needed about the relevance of anatomy in addressing medico–legal questions in clinical/surgical contexts. *Forensic Clinical Anatomy* has been defined as “the practical application of Clinical Anatomy to the ascertainment and evaluation of medico-legal problems”. The so-called *individual anatomy* (normal anatomy, anatomical variations, or anatomical modifications due to development, aging, para-physiological conditions, diseases, or surgery) may acquire specific relevance in medico–legal ascertainment and evaluation of cases of supposed medical malpractice. Here, we reviewed the literature on the relationships between anatomy, clinics/surgery, and legal medicine. Some methodological considerations were also proposed concerning the following issues: (1) relevant aspects of individual anatomy may arise from the application of methods of ascertainment, and they may be furtherly ascertained through specific anatomical methodology; (2) data about individual anatomy may help in the objective application of the criteria of evaluation (physio–pathological pathway, identification–evaluation of errors, causal value, damage estimation) and in final judgment about medical responsibility/liability. Awareness of the relevance of individual anatomy (risk of iatrogenic lesions, need for preoperative diagnostic procedures) should be one of the principles guiding the clinician; medico–legal analyses can also take advantage of its contribution in terms of ascertainment/evaluation.

## 1. Introduction

Although anatomical issues are intrinsically included in medico–legal methodology, higher awareness would be needed about the relevance of anatomy in addressing medico–legal questions in clinical/surgical contexts. Thus, in order to stress the opportunity for anatomical deepening in legal medicine, we recently proposed the term “Forensic Clinical Anatomy”, defined as “the practical application of anatomical knowledge and methods (from ultrastructural to macroscopic aspects), endowed with substantial clinical/surgical implications, to the ascertainment and evaluation of medico–legal problems” [1,2]. Specific symposia on Forensic Clinical Anatomy were organized in the context of the Intersocietal Symposium of the International Academy of Legal Medicine (Venice, June 2016) and of the Meetings of the International Federation of Associations of Anatomists (IFAA) (London, August 2019; Istanbul, August 2022). Issues of Forensic Clinical Anatomy may acquire relevance in various fields of legal medicine (child abuse, sudden death, personal injury, and damage), but in medical malpractice, forensic implications of clinical anatomy are more frequent and more significant.

In the second half of the 20th century, litigations for presumed malpractice have dramatically increased, particularly in Europe and the US [3]. In a survey about malpractice claims received by a large liability insurer covering physicians in all of the US (period 1991–2005), each year, 7.4% of all physicians had a claim and 1.6% had a claim leading to payment [4]. In this study, it was estimated that by the age of 65 years, 99% of physicians in high-risk specialties had faced a malpractice claim. The specialties with a higher proportion of physicians having a claim each year were neurosurgery (19.1%), thoracic-cardiovascular surgery (18.9%), and general surgery (15.3%). A further study with data from the National Practitioner Data Bank (US, period 2005–2014) identified the specialties with a higher risk of claim recurrence (i.e., physicians receiving more than a claim) in neurosurgery (hazard ratio 2.32 with respect to internal medicine), orthopedics (2.02), general surgery (2.01), plastic surgery (1.95), and obstetrics–gynecology (1.89) [5].

Malpractice and clinical forensic medicine are among the main arguments addressed by medico–legal scientific research. A recent survey showed that the total number of papers about malpractice and clinical forensic medicine were 8.6% and 6.9%, respectively, of papers published by European authors between 2005 and 2010 in journals within the JCR category “Medicine, Legal” [6]. These themes, however, are mainly addressed in not specifically medico–legal journals. In another survey examining European medico–legal articles published in a 5-year-period (2005–2010), the percentages of articles about malpractice and clinical forensic medicine which were published in not specifically medico–legal journals were 86.9% and 64%, respectively [7]. The implications of forensic clinical anatomy may be mainly found in medico–legal articles about malpractice and clinical forensic medicine, although many papers about forensic pathology (22.1% of published papers in “Medicine, legal” journals) may also include anatomical considerations.

In the literature on medical malpractice, however, few papers have fully addressed the three dimensions of forensic clinical anatomy, through a rigorous analysis of *anatomical* variability/individuality in consideration of both *clinical/surgical* and *forensic* implications. Conversely, in the anatomical studies and meta-analyses of the last decades, the clinical/surgical implications of variability are usually addressed, but medico–legal considerations are almost always lacking. Thus, the aims of the present work were: (1) to increase the awareness (in anatomists, physicians/surgeons and forensic practitioners) about correlations between anatomy, clinical/surgical disciplines and legal medicine, through a series of examples from the literature and our professional experience; (2) to focus some methodological considerations about, (a) how to consistently identify relevant anatomical data in the ascertainment phase of analysis of medical responsibility hypotheses, and (b) how to correctly discuss them in the evaluation phase to achieve the final medico–legal judgment.

The articles for this narrative review were chosen through a screening of abstracts in the PUBMED and SCOPUS databases, using the following search terms in the title, abstract and keywords: “Anatomical Variations” OR “Clinical Anatomy” AND “Legal” OR “Forensic” OR “Medical Liability” OR “Medical Responsibility” OR “Malpractice” OR “Iatrogenic”. Bibliographies of selected papers were examined and cross-referenced for further literature.

## 2. Individual Anatomy in Clinical/Surgical and Forensic Settings

**Figure 1:**
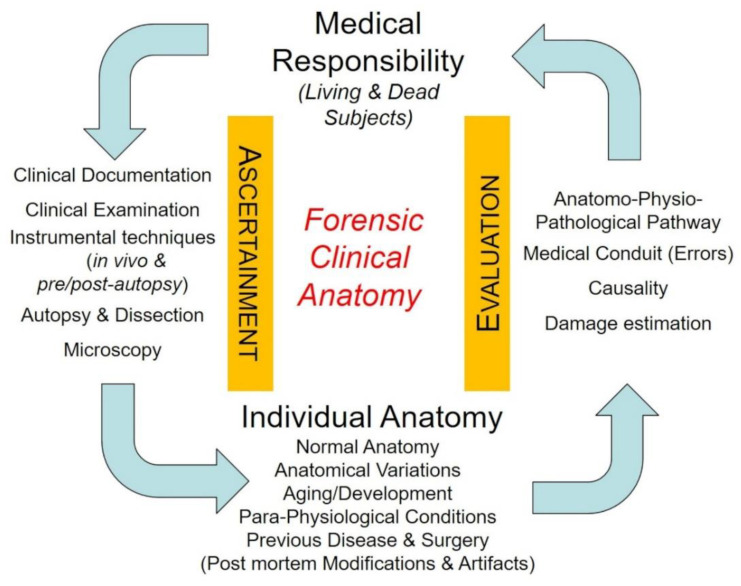
Forensic clinical anatomy and medical malpractice hypotheses. The individuality of anatomy is derived from ascertainment methods and must be considered in the evaluation phase for final judgement on medical responsibility/liability.

In the analysis of medical malpractice hypotheses, the “individuality” of anatomy acquires specific relevance. We have defined “individual anatomy” as “the anatomy of that specific person considered in the particular moment of clinical and/or forensic relevance”. The individual anatomy may be the *normal* “textbook” anatomy or it may be *variant*, on a congenital basis (the so-called anatomical variations), or *modified* as the result of modifications due to development, age, para-physiological conditions, disease, or surgery [1,2,8] (Figure 1). The “individuality” of the patient is actually also stressed in some surgical guidelines for its implicit legal implications. The European Society for Vascular Surgery, for instance, clearly states that its guidelines on the management of abdominal aorto-iliac artery aneurysm “under no circumstance should […] be seen as the legal standard of care in all patients”; “the document provides a guiding principle, but the care given to an individual patient is always dependent on many factors including symptoms, comorbidities, age, level of activity, treatment setting, available techniques and other factors” [9].

As it regards anatomical variability, there is probably a lack of awareness about the prevalence of anatomical variations, their clinical/surgical implications (increased risk of iatrogenic lesions, need of preoperative diagnostic procedures, …) and their role in the medico–legal analysis (ascertainment and evaluation) of supposed malpractice cases. Here, we present the clinical/surgical and forensic implications of some high-prevalence anatomical variations which invest the specialties with higher claims.

Two US surveys have been recently published about liability claims in neurosurgery [10,11]. In both studies, most claims involved spinal procedures, and improper performances were the most commonly cited reasons for litigation. An example of anatomical variation potentially investing in spine surgery is the foramen arcuale. It is a bony bridge located on the posterior arch of the atlas which encircles the vertebral artery. In a recent meta-analysis, the overall pooled prevalences of complete and incomplete foramen arcuale were 9.1% and 13.6%, respectively [12]. This variation increases the risk of (potentially fatal) injuries to the vertebral artery during the C1 lateral mass screw insertion for the treatment of atlantoaxial instability, as it can be overlooked and it can give a false impression of a broader posterior arch (e.g., [12,13]). It may be difficult to identify a foramen arcuale intraoperatively so that preoperative screening with CT has been “strongly” suggested as the gold standard for foramen arcuale identification; lateral radiographs show lower sensitivity with respect to CT and cadaveric dissections [12]. On the basis of the above clinical anatomy data, medico–legal implications in the case of vertebral artery injury due to un-identified foramen arcuale are clear (omitted or misdiagnosed pre-surgical imaging? Anatomically incongruent surgical procedure?).

Hepatic–bilio–pancreatic surgery has been reported to be one of the surgeries showing a higher proportion of medical liability claims in general surgery [14]. Complications frequently involve injuries to the biliary and vascular structures, which are known to be frequently variant. For instance, in an operative series of 300 laparoscopic cholecystectomy anatomical variations or congenital anomalies were found in 10.67% of cystic arteries (arising above Calot’s triangle; coursing anterior, posterior or on the right of the cystic duct; double, aberrant, or short), 4.33% of cystic duct (short, long, or accessory cholecysto-hepatic duct), 2.67% of right hepatic artery (Moynihan’s hump anomaly), 2% of gallbladder (buried, floating, or phrygian cap gallbladder; parallel to common bile duct) and 0.67% of the common hepatic artery (long and tortuous), for an overall prevalence of anatomical variations of 20.33% [15]. Intrahepatic vascular and biliary structures also show high variability, with obvious surgical implications in procedures such as anatomical liver segmentectomy. Anatomical variations of the portal vein branching have been reported in about 20% of cases (e.g., [16,17,18]). The typical pattern of intrahepatic bile duct ramifications has been reported only in 64.5% of cholangiograms of a recent European series and in 60.8% of cases derived from a large meta-analysis work [19]. Variant anatomy may increase the risk of iatrogenic injury but cannot be considered a cause of justification per se.

Total hip arthroplasty is one of the more frequent interventions in orthopedics. In a Dutch survey of hip arthroplasty malpractice claims, sciatic nerve injury was the most frequent reason for a claim (19.6% of all claims) [20]. In a large series of total hip arthroplasty, Navarro et al. [21] reported that anatomical variations were the primary cause of postoperative nerve palsies. In particular, the normal exit of the sciatic nerve from the pelvis is as a single entity below the piriform muscle; a recent meta-analysis, however, reported that its course is variant in 14.8% of cases, the most frequent variation (9.8%) involving the common peroneal nerve piercing the piriform muscle and the tibial nerve passing below it [22]. Variant anatomy of the sciatic nerve is known to increase the risk of iatrogenic palsy after total hip arthroplasty. For instance, tension-related injuries are more frequent if the sciatic nerve or one of its components passes through the piriform muscle: tenotomy of the piriform muscle, if required, may produce an anomalous strain to the nerve. The posterior approach shows a higher risk of nerve injury also due to the frequent requirement to detach the external rotators, such as the piriform muscle [23]. In the literature, medico–legal implications of nerve palsy after hip replacement have been discussed but without specific considerations about the importance of anatomical variability, apart from reference to the need of “a thorough awareness of the surgical anatomy of the region” [24].

Sometimes anatomical variability may also face different terminologies used by different disciplines. An example is the anal canal and its cranial limit. The so-called “anatomical” (or “embryologic”) anal canal goes from the anal verge to the dentate (or pectinate) line, which is the limit between non-keratinized squamous epithelium and simple columnar epithelium. The anal transitional zone extends cranially to the dentate line as a zone of gradual transition. The”surgical” (or “functional”) anal canal extends from the anal verge to the anorectal ring, which is palpable as a tight ring-like structure and it is given by the fusion of the puborectalis part of the levator ani with the internal and external sphincter muscles. Hemorrhoids are sub-divided into internal and external with reference to their location above or beneath the dentate line, respectively. Different surgical approaches are available, which rely on specific anatomical landmarks for complication prevention. For instance, for internal hemorrhoids, stapled hemorrhoidopexy is a circumferential resection of rectal mucosa which is performed superiorly to the dentate line; if the suture extends to the dentate line, chronic anal pain may arise as a complication, as the squamous epithelium of the anatomical anal canal is highly innervated by sensitive terminals [25,26]. Moreover, proctalgia and bleeding are also associated with the presence of muscle fibers in the excised tissue [27]. Thus, anatomical variability in levels of the dentate line or in the wall thickness of the anal canal may increase the risk of complications.

The anatomy of each specific person also changes along the whole lifespan so that development- and age-related modifications must also be considered by physicians/surgeons and medico–legal practitioners.

Some para-physiological conditions may also affect and modify the normal anatomy, although they are not always sufficiently considered in clinical/surgical settings and in medical malpractice analyses. *Pregnancy* obviously induces a series of anatomical modifications involving not only the uterus but potentially all the anatomical structures. For instance, anatomical changes which occur in the breast can potentially be misdiagnosed as pathological [28]. Apart from obvious effects on skeletal musculature, *sport training* may also produce less apparent anatomical and functional modifications, which may be underestimated. For instance, exercise-induced enlargement of cardiac chambers is commonly observed in competitive athletes, and some authors have defined the normal limits of biventricular size and function estimated by cardiac magnetic resonance in competitive athletes, these normal reference values being to be considered as an alternative to standard upper limits derived from the general population when interpreting images in athletes [29]. Conversely, an athlete’s heart and hypertrophic cardiomyopathy may be misdiagnosed and they potentially may coexist, thus putting problems in terms of differential diagnosis and medical responsibility [30].

Apart from anatomical variations, a series of pathological conditions may modify the topographic relationships of otherwise normal anatomical structures (pathology-modified anatomy). All the structures or organs characterized by a volumetric increase (malignant or benign neoplasm, …) may modify the topographic anatomy of the region, with frequently underestimated clinical implications. For instance, abdominal masses from the uterus or ovaries may produce direct contact with the lumbosacral trunk; in these cases, the lumbosacral trunk, although not pathological per se, must face a modified topographical anatomy which may imply sciatica secondary to nerve compression. It has been stressed, in the literature, that in order to prevent misdiagnosis and ineffective spine surgery, gynecological/obstetrical causes of sciatica-like symptoms must be kept in mind and addressed [31]; in other words, physicians must always consider possible anatomical changes induced by pathology.

It is widely known that surgery-induced modifications of normal anatomy may increase the risk of iatrogenic injuries in following operations or revisions (surgery-modified anatomy). For instance, the nerve palsy rate has been reported to be 1.3% after hip primary replacement and 3.2% after revision surgery [32], as nerves embedded in dense scar tissue have a lower nerve blood supply and show a lower capability to be stretched without damage [24,33,34]. Peritoneal adhesions due to previous surgery also frequently modify the topographic relationships between visceral, vascular, and nervous structures, with increased risks of iatrogenic injuries. We have previously reported an iatrogenic lesion of the femoral nerve due to trocar insertion for laparoscopic ovariectomy. Although a too caudal insertion in the iliac fossa is permitted to identify a medical error, the role of peritoneal adhesions due to previous gynecologic surgery was also discussed. In particular, adhesions reduced the peritoneal safety space between the abdominal wall and femoral nerve, increasing the risk of nerve injury due to inadequate pneumoperitoneum and poor visualization [35].

Finally, in autoptic cases, it is frequently important to consider if some anatomical or pathological data are ‘vital’ or due to post-mortem transformative modifications or technical artifacts. For instance, it is widely known that some hypostases may be misdiagnosed as bruises or ecchymoses. In a series of 230 forensic autopsies, Sauvageau and Racette [36] reported that post-mortem artifacts misinterpretation occurred in 7.83% of all requested forensic autopsies and in 35.29% of decomposed autopsies. It has also been reported that embalming procedures may produce alterations of the aortic morphology, which may mimic aortic dissections, not only on gross inspection but even on post-mortem imaging [37]. In pre-autopsy imaging, radiologists without specific experience may also misdiagnose some anatomical data due to post-mortem modifications (see later for example).

The most recent evolution of doctrine in legal medicine has stressed the importance of personalization in clinical practice (personalized medicine) and forensic settings (personalized justice) (e.g., [38,39]). In particular, the term “personalized medicine” was created by the US National Cancer Institute for healthcare addressing information on individual genomes, proteins, and environment for diagnosis and treatment [40]. In personalized medicine, the relevance of high-throughput technologies has been emphasized for the so-called “omics revolution”, comprising genomics, transcriptomics, proteomics, and metabolomics, possibly integrated in a view of systems biology [38].

The concept of individual anatomy may also be considered one of the prerequisites of personalized medicine, with consequent implications in personalized justice, also considering the huge mass of anatomical data which can be derived from the most modern imaging techniques (radiomics, [41]).

## 3. Clinical Anatomy in Medical Malpractice Analysis—Methodological Issues

Further methodological considerations may be formulated about how individual anatomy, according to the above definition, must be critically considered along ascertainment and evaluation phases of analyses of medical malpractice hypotheses, i.e.,

(1) how aspects of individual anatomy (of relevance from a forensic point of view) may arise from the application of methods of ascertainment, and how they may be furtherly ascertained through specific anatomical methodology;
(2) how data about individual anatomy, once fully ascertained and consistently discussed in the light of pertinent scientific knowledge, may help in the correct application of the criteria of evaluation and in final judgment about the identification of profiles of medical responsibility/liability.

Obviously, issues of forensic clinical anatomy, with consistent methods of ascertainment and criteria of evaluation, must be fully integrated into the methodology of medico–legal analysis of medical responsibility and/or liability. Medical malpractice legislation is quite different among nations and excellent review works are available on the matter. However, it has also been stressed that, even in different normative contexts, the methods of ascertainment and criteria of evaluation should be shared on the basis of scientific principles.

### 3.1. The Relevance of the Individual Anatomy in the Application of Methods of Ascertainment on Living Persons

Although the relevance of individual anatomy may be overlooked, anatomical data of interest may arise from the various steps of ascertainment on living persons, and they may be furtherly detailed through additional focused analyses.

In the analysis of clinical documentation, for instance, it is important to preliminarily verify if physicians have explicitly identified anatomical elements of possible/probable clinical relevance (anatomical variations, development/age- or surgery/disease-related modifications) or if they have considered their potential presence, in terms of possibility or probability (for instance, possible/probable adhesions due to previous surgery). The medico–legal consultant must also verify if the clinician has associated the (ascertained or hypothesized) individual anatomy with modified risks. In this sense, the presence of explicit references to individual anatomy and associated iatrogenic risks in the informed consent documents could be verified.

A further step is the analytic examination of the clinical documentation in order to consider if other relevant anatomical findings (not focused on by the clinicians) can be identified. In this sense, imaging revision is of pivotal importance to verify if other anatomical individualities are present.

In anamnesis, communications by physicians to the patient will also be considered regarding the identification of relevant anatomical data and associated modified risks. Physical examination may permit direct identification of anatomical variations at the level of various structures, such as skin, bones, muscles, or natural orifices. For instance, anatomical variations of the larynx, such as a high-rising epiglottis, can be identified at physical examination [42]. Neurological examination may also permit the identification of variations in the territories of some nerves [2].

Apart from the identification of the anatomical structure injured, analysis of the damages and functional limitations may give further information about the preceding anatomical situation. For instance, the consequences of a nerve iatrogenic injury may be very different in terms of sensory and/or motor impairment on the basis of territory innervation, which can be widely variant.

Medical malpractice cases may also be addressed with dedicated imaging examinations if the available data are not sufficient for detailed diagnosis. This may also be possible if the existence of a relevant individual anatomy derives from clinical/documental data and/or clinical examination (or it may be hypothesized on their basis), but further detailing is needed [2]. Similarly to post-mortem imaging (see later), some authors have stated that malpractice cases with suspected vascular lesions would potentially benefit of angiographic methods [43]; analogous considerations could be formulated for detailing the vascular anatomy of the district. Specific evaluations must be performed, however, about the feasibility of imaging techniques from an ethical and juridicial view, if in a purely forensic context without clinical indication [43].

Other instrumental techniques may also be useful in the identification of anatomical data of relevance. Electromyography, which is frequently involved in the analysis of iatrogenic nerve lesions, may also add data about nerve territories and innervation of normal and variant muscles. For instance, electrophysiological tests have been used to investigate in vivo the percentage of variant innervation of the first dorsal interosseous muscle by the radial nerve [44]. Snoek et al. [45] used surface stimulodetection electromyography to study in vivo the innervation of the axillary arch, a muscular anatomical variation of the axillary region. They proved that this muscle receives the same innervation of the latissimus dorsi (thoracodorsalis nerve), stressing that this technique is a practical and reliable tool “for investigating anatomical aspects of muscle innervation in vivo”. Conversely, the anatomical potentiality of electromyography may be undervalued in clinical and forensic contexts.

### 3.2. The Relevance of the Individual Anatomy in the Application of Methods of Ascertainment on Dead Persons

#### 3.2.1. Examination of Clinical Documentation

In the analysis of pre-clinical/clinical data for deaths due to supposed medical malpractice, it is important to preliminary verify all the data which could be useful in the planning of following ascertainment steps, in particular eventual need of pre-autopsy imaging examination and specific protocols of an autoptical approach. This is particularly true if anatomical findings arise that need dedicated (instrumental or autoptical) anatomical methods.

**Figure 2:**
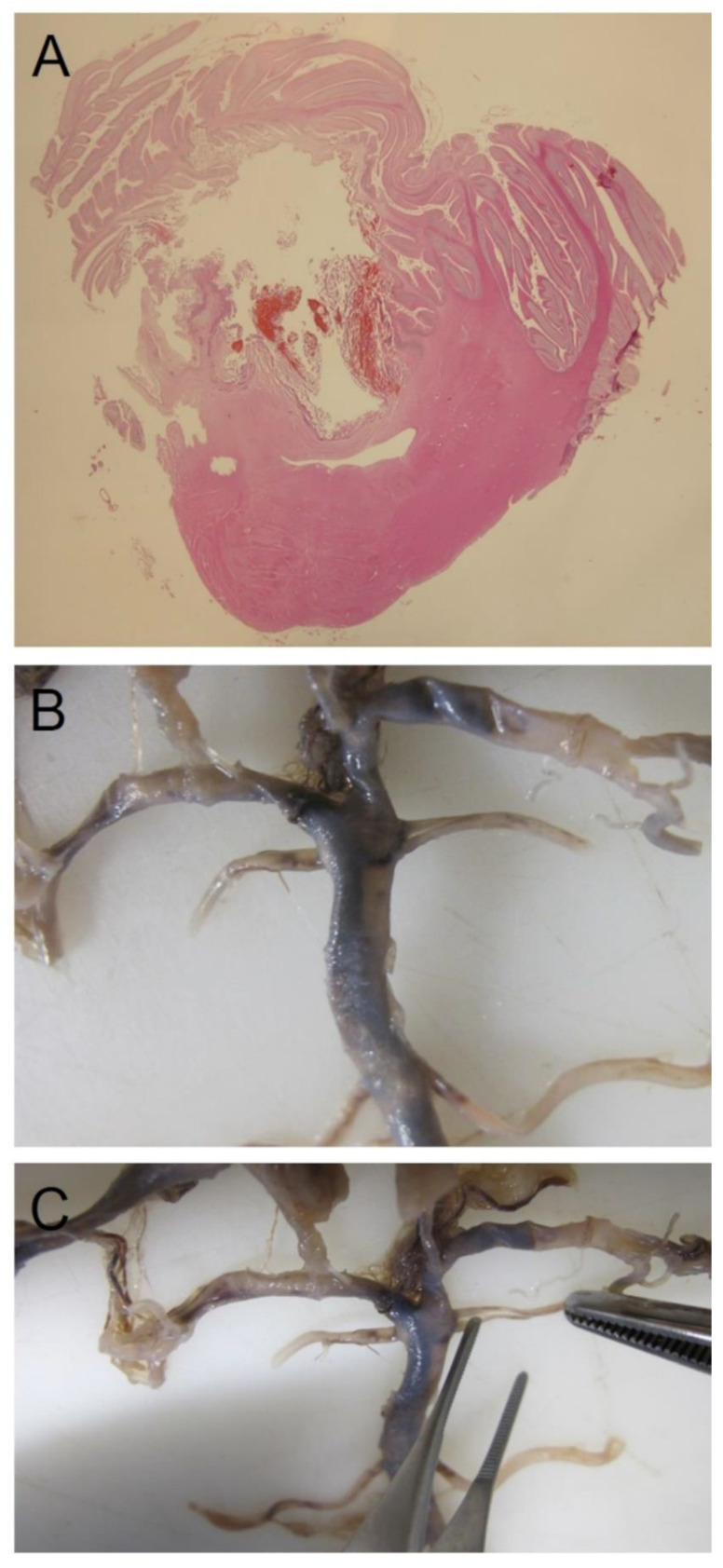
(**A**) Cerebellar macro-section showing hemorrhagic infarction in the vascular field of the superior cerebellar artery. (**B**,**C**) Double origin of the left superior cerebellar artery at macroscopic examination of the vessels of the brain.

Imaging revision could also be needed, even if it is frequently performed in integration with autopsy findings. As it regards the importance of imaging revision (before autopsy or in integration with autopsy findings), we reported a case of a cerebellar hemorrhagic–ischemic lesion in which in vitam CT angiography reported “probable double left superior cerebellar artery” whereas imaging revision (confirmed by following neuropathological examination) permitted to identify a”double origin” of the superior cerebellar artery [46] (Figure 2).

#### 3.2.2. Pre-Autopsy Examinations

Many different imaging techniques are available for pre-autopsy examinations, such as conventional X-rays, CT, MR, post-mortem angiography, multi-phase post-mortem CT angiography, 3D surface scanning/photogrammetry, integrated CT/MR and surface scanning (reviewed in [47,48,49,50,51,52,53,54]). In forensic clinical anatomy, pre-autopsy imaging techniques are obviously of pivotal importance, as they can lead to the identification of a previously unknown “individual anatomy” of potential forensic interest or they can be specifically used to clarify in detail a variant or modified anatomical situation already derived from the clinical data.

Post-mortem CT shows higher sensitivity than an autopsy for detecting bone lesions, also because some districts are not usually accessed during autopsies [54]. Similarly, post-mortem CT may identify previously unrecognized anatomical variations or age-related changes with possible implications in cases of medical malpractice.

Post-mortem MR is usually used less frequently than post-mortem CT due to limited access and complexity. However, post-mortem MR is more accurate than CT for the analysis of soft tissues and abdominal organs [54]. As regards the evaluation of anatomical findings in the forensic context, it proved useful to have a preliminary measurement of the thickness of ventricular walls [50]. Moreover, post-mortem MR performs better than CT in children, due to reduced abdominal and subcutaneous fat and poor soft tissue contrast in the brain [55], being potentially useful for assessing developmental aspects of anatomy.

Post-mortem CT angiography has been used in forensic radiology for whole-body approaches (i.e., [56]) and selective analyses of coronary arteries [57,58]. The most recent evolution is the multi-phase post-mortem CT angiography, which includes a native CT scan followed by further CT-acquisitions after contrast injection, in arterial, venous, and dynamic phases (i.e., [59]). Multi-phase post-mortem CT angiography proved particularly useful in the anatomical identification of bleedings, with obvious usefulness in medical malpractice cases [60,61]. Postoperative hemorrhage represents one of the most common complication after surgery [62]. In the literature, comparative analyses are also present between PMCT, PMCTA and autopsy [63,64]. For complicated cases of supposed medical malpractice after surgical interventions, an integration between conventional CT, MPMCTA and autopsy has been proposed as the gold standard for post-mortem investigation (i.e., [60]).

Multi-phase post-mortem CT angiography is obviously particularly useful for the identification of anatomical variations and evaluation of their clinical and forensic implications. For instance, anatomical variability in the cervical loop segment of the vertebral artery has been studied with post-mortem CT angiography to evaluate its potential predisposing effect for basal subarachnoid hemorrhage [65]. Zerlauth et al. [60], in a case of hemorrhagic shock after splenectomy, identified a variant bifurcation of the splenic artery and the source of hemorrhage in one of the branches which were not ligated at surgery. In this case, the anatomical variation was not visible at conventional CT and it was identified through dissection only on fixed tissue and after revision of the angiographic images. This report, together with indirect evidence from the above comparative studies, supports a higher sensitivity of MPMCTA for detecting vascular anatomical variations. In fact, post-mortem CT angiography has also been used for “conventional” anatomical studies of various vascular districts (i.e., [66,67]).

Sabatasso et al. [68] also stressed the advantages of applying multi-phase post-mortem CT angiography before autopsy to investigate the “modified vascular anatomy” as a consequence of previous vascular by-passes. The usefulness of whole body post-mortem CT angiography has also been demonstrated in a 37-week-old fetus following in utero fetal death [69], suggesting a potential role of this technique in appreciating the developmental aspects of vascular anatomy.

The technical feasibility of post-mortem MR angiography has also been demonstrated, allowing the soft tissue detail of MR and the vascular details of angiography [50,70]. However, the literature on the matter is more limited.

**Figure 3:**
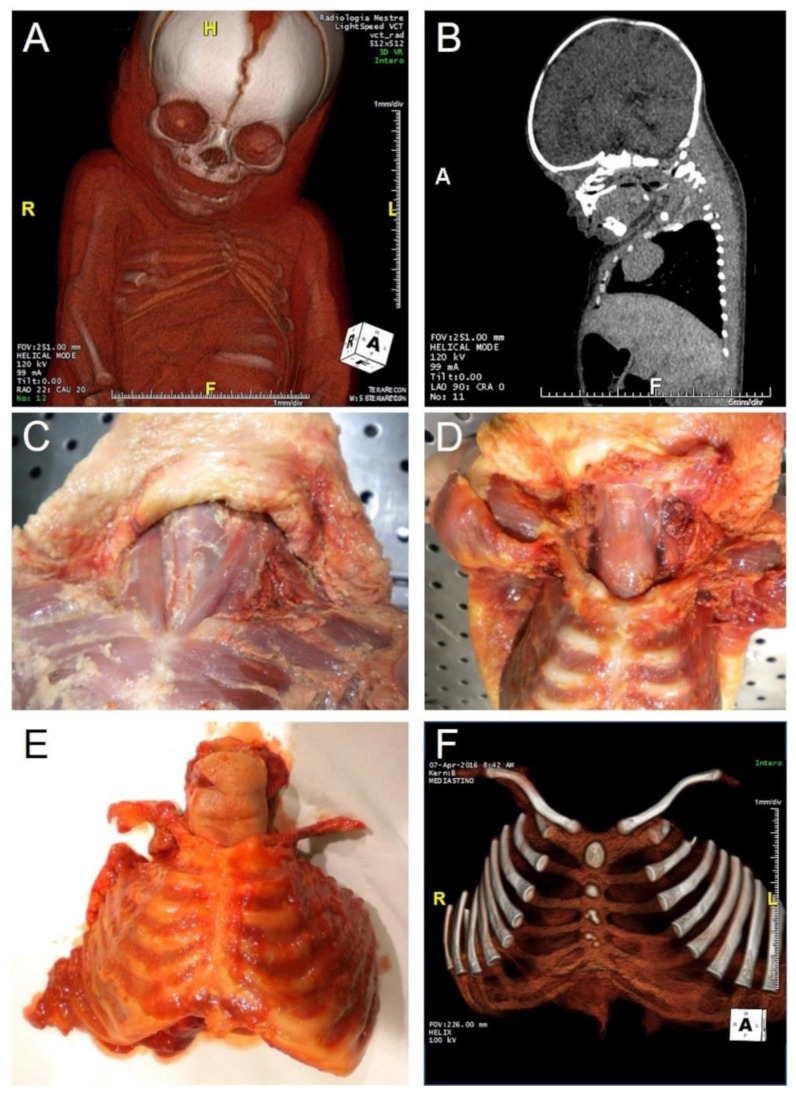
Infant reported to be found dead in the parents’ bed: accidental or abusive trauma? Sudden Infant Death Syndrome? Undiagnosed pathology? (**A**,**B**) post-mortem computed tomography, with radiologist’s description of thorax deformation. (**C**,**D**) anatomical dissection of the neck and thorax, showing the absence of hemorrhagic infiltrations or fractures/dislocations. (**E**) Sampling of the whole anterior thoracic wall. (**F**) post-autopsy computed tomography of the anterior thoracic wall, confirming the absence of traumatic findings. The radiological misdiagnosis was ascribed to artifacts due to previous corpse freezing in an anomalous position. (H: Head; F: Feet; L: Left; R: Right).

Many authors have also stressed the importance of modifications of normal radiologic anatomy due to post-mortem transformative changes or artifacts (fluid redistributions, position-dependant sedimentations, changes in the inter-tissutal natural contrast on T1 and T2 weighted images), which can represent pitfalls (differential diagnosis between anatomy and pathology) for clinical radiologists without specific experience on post-mortem investigations [60,71]. In our experience, we performed pre-autopsy CT in a sudden infant death case in which hypotheses arose of undiagnosed pathology, sudden infant death syndrome, and accidental or abusive trauma. The radiologists described a series of potentially traumatic thoracic findings (deformation of the thorax for dislocation of the right sternoclavicular joint and flattening of the cranial right ribs; discrepancy between the second rib and the sternum; asymmetry of the right pectoral muscles with respect to contralateral ones) which were then excluded at autopsy and post-autopsy additional CT. The radiological misdiagnosis was ascribed to artifacts due to previous corpse freezing in anomalous position (Figure 3). In this sense, the need for specialists in the interpretation of post-mortem radiological investigations (“necroradiologists”) has also been recognised [71,72].

#### 3.2.3. Autopsy

It has been widely stressed that it is extremely important to properly conduct autopsies to ascertain cause of death in forensic cases. For instance, the role of autopsy is pivotal in sudden deaths [73]; in these cases, anatomical anomalies may also play a role, such as congenital coronary artery anomalies or valvular diseases. The recent COVID-19 pandemic furtherly stressed the clinical and epidemiologic implications of autopsies, which remain the gold standard method for ascertainment of cause of death [74,75,76]. In the first pandemic period, although the World Health Organization suggested post-mortem examinations following recommended safety procedures [77], some Governments, including the Italian one, discouraged autopsies in COVID-19 deaths [78]. In some reports, only partial autopsies were performed, sampling only the lungs [79] or performing only core biopsies of the main organs (lung, liver, heart) [80]. Complete autopsies, instead, also including the (initially neglected) brain, permitted to clarify the the extent of SARS-CoV-2 invasion and the pathophysiology of COVID-19, which may be considered a systemic disease affecting various organs and systems.

Many guidelines and best practices are available about forensic autopsy. The “Recommendation no. R (99) 3 of the Committee of Ministers to Member States” is specifically devoted to the “Harmonisation of Medico–legal Autopsy Rules” [81]. The European Council of Legal Medicine also published an accreditation/certification procedure about post-mortem investigations in forensic pathology [82]. Specific overviews have also been published about the forensic pathological approach in iatrogenic deaths (e.g., [83]). In this shared methodological context, further considerations may be proposed about the cases endowed with specific anatomical implications.

Judicial autopsies and anatomical dissections are characterized by different aims and methods: anatomical dissections are focused on detailing normal or variant anatomical structures; judicial autopsies are mainly focused on the injuries and damage. However, in cases endowed with forensic clinical anatomy implications, the specific analysis of the individual anatomy is necessary to reconstruct the physio-pathological pathway, to analyse the clinical conduit, to address causality and/or to correctly estimate damage. Thus, the ‘classical’ methods of the judicial autopsy must sometimes be integrated with anatomical dissections. As previously stated, the need of ‘pure’ anatomical data, to be investigated at the dissection table, frequently derives from preliminary analysis of clinical documentation or pre-autopsy imaging. The opportunity to modify procedures must always be considered, even in the course of autopsy, in response to new relevant data. For instance, iatrogenic lesions to vascular or nerve structures usually imply the need of anatomical dissections aimed to identify the injured structure; we would like to stress, in addition, that the anatomical pattern should also be considered, comprehensive of possible variants and modifications due to age, disease, surgery, and other factors.

Modified anatomy due to previous surgery also make dissection difficult for hemorrhagic infiltrations or fibrotic reactions, as stressed by various forensic pathologists (i.e., [60]). In these cases, the autopsy technique can be “anatomically” also adapted on the basis of preliminary data from post-mortem radiology.

Apart from the above methodological considerations, it is also important to stress that differential diagnosis issues may arise at autopsy between variant anatomy and pathology. Few papers have focused on this aspect. Advenier et al. [84], for instance, reviewed anatomical variations or malformations of the larynx, which could be misinterpreted as sequelae of old injuries or iatrogenic lesions.

#### 3.2.4. Post-Autopsy Imaging Techniques

**Figure 4:**
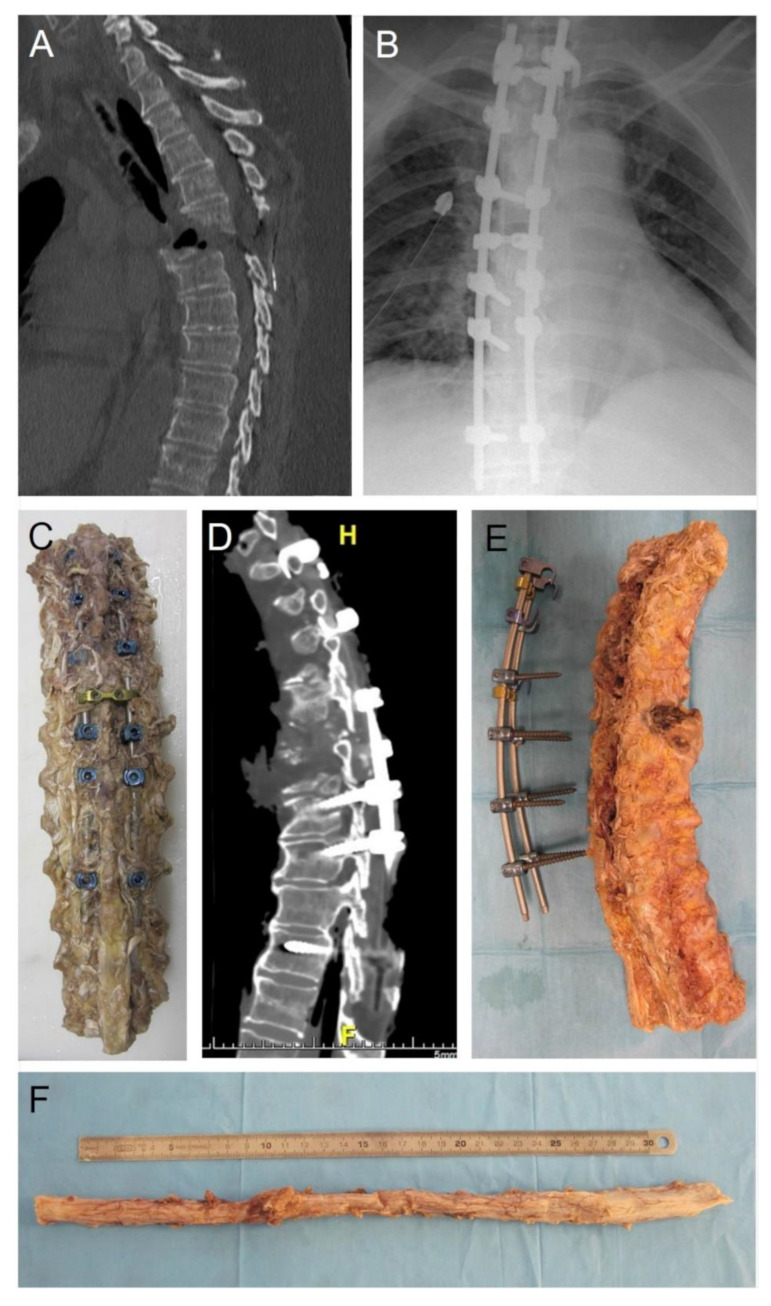
Spine trauma. (**A**) in vivo pre-surgery computed tomography. (**B**) in vivo post-surgery radiography. (**C**) sampling of the spine segment. (**D**) post-autopsy computed tomography of the sampled spine. (**E**) removing of pedicle screws. (**F**) general view of the spinal cord, with evidence of hemorrhagic infiltration of the dural sac. (H: Head; F: Feet).

Further diagnostic procedures may be applied to organs or structures sampled at autopsy. Post-mortem imaging applied to single organs or structures (X-ray, CT, MR, angiography, eventually with casts) is frequently involved in forensic pathology and may also give further information about anatomy. Hindso et al. [85] demonstrated the accuracy of post-mortem CT for the measurement of the volumes of epicardial adipose tissue and myocardium in eviscerated hearts. In addition, post-mortem imaging of explanted larynxes after forensic autopsy have been proposed [86] and in a forensic case we involved post-mortem CT for better detailing of a laryngeal anatomical variation (differential diagnosis with traumatic finding) [87]. We also performed post-autopsy CT in large thoracic samples, such as the thoracic anterior wall (ribs and sternum) (Figure 3) and thoracic spine segment (Figure 4). In the latter case, post autopsy CT permitted a detailed anatomical analysis of pedicle screw insertions. In this sense, in our experience, it is pivotal the integration between in vivo, pre-autopsy and post-autopsy imaging, obviously together with autopsy dissections.

The use of Micro-Imaging techniques (micro-CT, micro-MR, ultrasound microscopy) in forensic analyses of post-mortem samples is also largely increasing (reviewed in [88]). The anatomical details of these techniques show obvious implications in terms of Forensic Clinical Anatomy. Micro-CT has been used to determine myocardial structural characteristics [89] and three-dimensional organization of vascularization (for instance, placental vasculature in [90]). Micro-MR can be used to measure and calculate a series of bone parameters (bone mineral density, bone volume density, bone surface density, trabecular pattern factor, structural mean index, and connectivity density) [91,92], which objectively describe age-related modifications. Micro-MR may also allow the analysis of age-related anatomical rearrangements of hippocampal structures (reduction of the extended Ammon’s horn relative volume and increase of dentate gyrus relative volume) [93].

#### 3.2.5. Microscopic, Ultrastructural and Molecular Analyses

Apart from ‘classic’ histopathological analyses, we would like to stress that microscopic techniques are also pivotal to better detail some anatomical findings of potential clinical and forensic relevance. The normal microscopic anatomy of different anatomical structures may explain different risks of iatrogenic injuries. For instance, it is known that the common peroneal nerve is more vulnerable than the tibial nerve to compressive damage because it shows tightly packed fascicles rather than abundant connective tissue inter-mingled (e.g., [34]).

Moreover, apart from differential diagnosis with pathological or traumatic findings, microscopic analysis is pivotal to define developmental and age-related changes in various anatomical structures. In addition, microscopic techniques may demonstrate anatomical modifications induced by diseases or previous surgery; we can recall, for instance, the fibrotic reactions possibly enveloping nerves after surgical approaches, with the aforementioned increased risk of nerve palsy following surgical revision.

Three-dimensional reconstruction of microscopic findings through image-analysis software may also permit to better detail anatomical data of forensic significance, as we have performed in our experience to detail anatomical bases of hypoxic-ischemic injuries [94], disorganization in the three-dimensional disposition of the connective tissue of carotid bodies in opiate-related deaths [95], the human area postrema [96] and a stab wound pathway in heart ventricle wall [97].

Genetic findings may also integrate “classical” anatomical data, possibly suggesting underlying genetic syndromes, both on living and dead persons. For example, genetic testing for Marfan syndrome may be suggested by an increased diameter of the aortic root.

### 3.3. Evaluation Criteria and Individual Anatomy

In the well-acquainted methodology of medico–legal analysis of supposed medical malpractice, the main evaluation criteria to be considered are the following: reconstruction of the physio–pathological pathway; identification–evaluation of errors; discussion of causal value; damage estimation [98,99]. The individual anatomy may acquire specific relevance in each of the above criteria.

#### 3.3.1. Reconstruction of the Physio–Pathological Pathway

In medical malpractice, anatomical data frequently acquire specific relevance for proper definition of the physio–pathological pathway, concept which could be widened in anatomo–physio–pathological pathway. In this sense, the potential role of individual anatomy (normal, variant, modified) in realizing the final damage (chronic pathological state, permanent injury, death) needs to be discussed and evaluated [1,2].

In a forensic clinical anatomy context, the most common situation is the iatrogenic lesion of an anatomical structure (vessel, nerve, organ, …) during a diagnostic or therapeutic procedure. Apart from the anatomical identification of the injured structure, what is most important in these cases is the evaluation of why that structure was injured. Where, exactly, the procedure was performed and how was the anatomy of the region: normal, variant or modified? For example, we can think of the iatrogenic lesion of a nerve, with a normal or variant course, as a consequence of an operative procedure in a specific anatomical district. It is not sufficient to state which structure has been injured but we must specify (if possible) the anatomical basis of the lesion, by comparison between the ‘anatomy’ of the procedure and the ‘anatomy’ of the structure itself. Disease- and surgery-related modifications (e.g., peritoneal adhesions, fibrotic reactions) may also increase the risk of iatrogenic injuries. These anatomical considerations, which are implicit to legal medicine, are not always fully addressed and may benefit of focused methodological approaches and dedicated research.

In other cases, a peculiar anatomical pattern may have a contributory role in producing a pathological state or it may modify the consequence of a lesion or disease. For instance, the above-mentioned double origin of the superior cerebellar artery probably played a role in the occurrence of a hemorrhagic cerebellar infarction, producing an anatomical situation of hemodynamic instability due to the small diameters of the two vessels [46]. Another example is the study by Papantchev and Colleagues [100] that classified the Willis circle variations, which could cause hypoperfusion during unilateral selective cerebral perfusion, finding potentially dangerous variations in 58.6% of cases. The injury of a nerve or the closure of a vessel may produce very different damages on the basis of the extent of the territory of innervation or vascularization.

#### 3.3.2. Identification-Evaluation of Errors

In the analysis of the medical conduit from a forensic clinical anatomy perspective, it is pivotal to verify if clinicians identified relevant anatomical data (or if they put the hypothesis explicitly or implicitly) and how they considered anatomy in the context of diagnostic, prognostic, and therapeutic procedures. It should also be identified what would have been the ideal medical conduit, integrating the ascertained findings, the reconstructed physio-pathological pathway and literature data on the matter. A thorough literature survey should address the following questions:

- are the relevant anatomical data (anatomical measurements or parameters, anatomical variations or modifications) described in the literature? And in which type of studies (case reports, case series, reviews, meta-analysis, …)?
- what is the reported prevalence (also with reference to different methods of ascertainment)?
- is it possible to identify the above anatomical data in the living person? And how (physical examination, imaging or other instrumental analyses, surgical intervention)?
- Are there instrumental or surgical procedures that are specifically required (rules of professional medical conduit) to clarify the individual anatomy, on the basis of the evaluation of risk-benefit ratios (complication risks, economic costs, …) of diagnostic procedures?
- If detailing of individual anatomy is not possible (absence of instrumental or surgical procedures), are there specific operative protocols to preserve anatomical structures, even in the presence of congenital or acquired variability?
- General “Anatomical” errors could be the following:
- omitted consideration of the anatomical data (may they be normal, variant, or modified) due to ignorance, error being more severe with increasing prevalence (and consequent foreseeability) of the anatomical pattern;
- omitted procedures able to identify the relevant anatomical data;
- inadequate management of an ascertained or supposed anatomical situation in terms of prognosis and therapy;
- omitted operative protocols aimed at preserving anatomical structures, particularly in the possibility of frequent anatomical variations or modifications.

Errors may also be made in the differential diagnosis between normal (or modified) anatomy and pathology. For instance, a critical issue endowed with forensic implications is the differentiation between para-physiologic structural remodeling of the athlete’s heart and cardiac abnormalities (reviewed, for instance, in [101]).

Iatrogenic lesions of normal or variant/modified anatomical structures may also derive from different types of anatomy-related errors which have been listed in Table 1 (with consistent literature examples [12,35,46,102,103,104,105,106]). It must also be considered that iatrogenic injuries may be caused by previously unreported and unidentifiable anatomical variants may be considered as no-fault medical accidents, due to the general absence of anatomical knowledge, adequate diagnostic tests and/or totally (anatomically) safe operative procedures. Although the relevance of normal and variant anatomy for iatrogenic nerve injuries is widely known, literature also addressing the forensic implications is quite scanty. For instance, Cesmebasi and Spinner [107] considered the anatomy of the spinal accessory nerve with reference to iatrogenic lesions and malpractice litigations.

A particular type of anatomy-related error is the so-called surgery at the wrong site. For instance, it has been widely stressed that spine anatomical variations (transitional vertebrae, rib variants, hemivertebrae, fused vertebrae) or modifications (tumors, infections, previous surgery, obesity, osteoporosis), if not correctly identified and managed, may cause errors in spinal enumeration and consequent spine surgery at the wrong level [104,105,106].

In a more general view, the importance of anatomical education and surgical training with cadavers from Body Donation Programs [108] has been widely stressed in the literature, and the forensic implications of insufficient/neglected operative anatomical education or training on cadavers have also been recently considered [109].

#### 3.3.3. Discussion of Causal Value

The identification and evaluation of what we can call “anatomy-related” errors imply a following discussion about their causal value, on the light of anatomo–physio–pathological pathway, possibly expressed in probability level, i.e., in terms of exclusion, possibility, probability or certainty. In the evaluation of the material causality in the cases in which anatomy plays a pivotal role, the relevance of the literature on the matter could be stressed in terms of anatomical measurements and parameters, the prevalence of anatomical variations and modifications, analysis of associated risks, the sensitivity of diagnostic procedures and prevention efficacy of operative protocols. In Table 1, a series of considerations/questions are listed about causality, which can be synthesized in the following question (counterfactual reasoning):

- if individual anatomy had been correctly identified, considered, and managed, the damage would have been prevented? And with what probability?

#### 3.3.4. Damage Estimation

Individual anatomy may determine the extent of the damage. For instance, a nerve injury could imply more severe damage if that nerve shows a wider territory of innervation for an anatomical variation. This aspect of evaluation, as already stated, mainly invests the biological aspects of damage, and should be included in the reconstruction of the physio-pathological pathway, with particular reference to the final clinical picture.

Further and different evaluation, instead, must address the quantification of damage (temporary and/or permanent biological injury) causally related to errors. In other words, the medico–legal consultant must specifically evaluate which part of the whole damage must be ascribed to an inadequate medical conduit. With reference to the anatomy of forensic relevance, it is possible that ignorance of an anatomical variation (anatomy-related error) may worsen a damage that would otherwise be lighter and (eventually) justified. The comparative evaluation must obviously be performed on the basis of specific anatomical considerations.

## 4. Conclusions and Future Perspectives

In the present work, the methods of forensic clinical anatomy [1,2] have been focused on the field of medical malpractice, both from general/research and particular/applicative points of view.

A first look at the literature on clinical anatomy has shown that, although the modern anatomy puts particular attention to the clinical/surgical implications (and frequently they represent the primum movens of anatomical research), forensic implications are frequently neglected. Conversely, surveys of medical malpractice rarely consider the clinical anatomy basis of medical conduit and causally related damages. This is the main limit of forensic clinical anatomy assessment, but it should also represent the stimulus for renowned research on the matter. Awareness about the forensic relevance of some clinically-oriented anatomical data may give further impulse to research on clinical/surgical anatomy and legal medicine.

From an applicative point of view, we have focused how relevant anatomical findings must be addressed in the medico–legal analysis of suspected cases of medical responsibility and/or liability, through the integration of methods of ascertainment with anatomical methods and discussion of evaluation criteria on the basis of critical review of clinical anatomy literature. In this sense, a difficulty in a forensic clinical anatomy approach is the little attention paid to the assessment of the individual anatomy in the clinical and forensic contexts, so that frequently few reliable anatomical data are present in clinical documentation and medico–legal reports by other consultants.

In the previous sub-paragraphs about methods of ascertainment on living and dead persons, we have discussed the role which clinical and post-mortem forensic imaging is playing in the evolution of the medico–legal sciences. It has been hypothesized that in the future, many other innovative imaging techniques and methods will be applied in the forensic field, such as functional MRI, tractography MRI, photoacoustic tomography/endoscopy, ptychographic X-ray CT, optical frequency domain imaging, spectral domain optical coherence tomography and FMT-XCT [105]. These methods (and, surely, others to come) will permit to furtherly detail the individual anatomy and its implications, in clinical and forensic contexts. The most recent evolutions of micro-imaging analysis (nano-radiology and integrations with chemical/molecular imaging) will probably extend their use in forensic (and forensic clinical anatomy) applications.

In our opinion, higher awareness and renowned interest about the strict correlations between anatomy, clinics/surgery and legal medicine (in a forensic clinical anatomy perspective) would represent a way to bridge gaps between disciplines with reciprocal advantages.

## Figures and Tables

**Table 1:** Iatrogenic lesions of normal/variant/modified anatomical structures: potential anatomy-related errors and considerations in terms of medical conduit and causality.

Potential Errors	Examples [References]	Graduation of Medical Conduit Inadequacy	Causality Questions with Reference to Individual Anatomy (Exclusion/Possibility/ Probability/Certainty)
Improper procedure execution	- Wrong site of gluteal intramuscular injection with lesion of the sciatic nerve [102] - Improper subclavian vein cannulation with injury of the brachial plexus [103] - Wrong site of trocar insertion with injury of the femoral nerve [35]	- Possibility of unpredictable anatomical variations/ modifications?	- Correct execution would have prevented the iatrogenic lesion?
Neglected identification of anatomical variation/modification at preoperative imaging	- Improper anatomical definition of a vascular anatomical variation (double superior cerebellar artery instead of the double origin of superior cerebellar artery [46])	- Difficulty of radiological analysis?	- Correct identification would have prevented iatrogenic lesion?
Neglected preoperative imaging	- Un-identified foramen arcuale due to omitted pre-surgical Computed Tomography [12]	- Protocol indications?	- Preoperative imaging would have permitted identification of anatomical variation/modification? - Following correct identification would have prevented iatrogenic lesion?
Improper anatomical localization due to variations/modifications and surgery at wrong site	- Wrong spinal enumeration and surgery at the wrong site due to anatomical variations (transitional vertebrae, rib variants, hemivertebrae, fused vertebrae) or modifications (tumors, infections, previous surgery) [104,105,106]	- Difficulty of radiological/ surgical anatomical analysis?	- Correct enumeration would have prevented damage due to wrong site surgery?
Omitted/delayed diagnosis/ prognosis/therapy of lesions of anatomical structures	- Undervalue of post-operative symptoms/signs and omitted/delayed/erroneous instrumental examinations for nerve injury (brachial plexus [103], femoral nerve [35], sciatic nerve [102])	- Difficulty of clinical/surgical/ radiological analysis?	- What would it be the damage if correct diagnosis/prognosis/therapy?

## Data Availability

Not applicable.
